# Comparison of Outcomes of Ischemic Stroke Initially Imaged With Cranial Computed Tomography Alone vs Computed Tomography Plus Magnetic Resonance Imaging

**DOI:** 10.1001/jamanetworkopen.2022.19416

**Published:** 2022-07-21

**Authors:** Heitor Cabral Frade, Susan E. Wilson, Anne Beckwith, William J. Powers

**Affiliations:** 1 Department of Neurology, the University of Texas Medical Branch at Galveston; 2 Department of Neurology, University of North Carolina School of Medicine, Chapel Hill

## Abstract

**Importance:**

Patients with acute ischemic stroke often undergo magnetic resonance imaging (MRI) in addition to computed tomography (CT), but its association with clinical outcomes is uncertain.

**Objective:**

To assess whether clinical outcomes of patients with acute ischemic stroke with initial CT alone were noninferior to those with additional MRI.

**Design, Setting, and Participants:**

A retrospective observational propensity score–matched cohort study of clinical outcomes at discharge and 1 year for patients hospitalized with acute ischemic stroke was conducted at an academic medical center between January 2015 and December 2017. Data collection from an electronic medical record system performed from May 2020 through January 2022 was not completely blinded. Noninferiority margins were based on the designs of previous randomized clinical trials of ischemic stroke treatments. Statistical analysis was performed in January 2022. Participants were adults hospitalized with acute ischemic stroke with admission diagnosis based on CT. Exclusion criteria were primarily missing data. From 508 eligible patients, all 123 cases with additional MRI were propensity-score matched to 123 controls without.

**Exposure:**

MRI after initial diagnosis.

**Main Outcomes and Measures:**

Death or dependence at hospital discharge (modified Rankin Scale score of 3-6) and stroke or death occurring in survivors within 1 year after discharge.

**Results:**

Among 246 participants, the median age was 68 years (IQR, 58-78.8 years) and 131 (53.0%) were men. Death or dependence at discharge occurred more often in patients with additional MRI (59 of 123 [48.0%]) than in those with CT alone (52 of 123 [42.3%]; absolute difference, 5.7%; 95% CI, −6.7% to 18.1%), meeting the −7.50% criterion for noninferiority. Stroke or death within 1 year after discharge determined for 225 of 235 (96%) survivors occurred more often in patients with additional MRI (22 of 113 [19.5%]) than in those with CT alone (14 of 112 [12.5%]; relative risk, 1.14; 95% CI, 0.86-1.50), meeting the 0.725 relative risk criterion for noninferiority.

**Conclusions and Relevance:**

This propensity score–matched cohort study of patients hospitalized with acute ischemic stroke found that a diagnostic imaging strategy of initial CT alone was noninferior to initial CT plus additional MRI with regard to clinical outcomes at discharge and at 1 year. Further research is needed to determine which patients hospitalized with acute ischemic stroke benefit from MRI.

## Key Points

**Question:**

Are clinical outcomes of patients with acute ischemic stroke imaged with initial computed tomography (CT) alone noninferior to those who had additional magnetic resonance imaging (MRI)?

**Findings:**

In this propensity score–matched cohort study of 246 patients hospitalized with acute ischemic stroke, a diagnostic imaging strategy of initial CT alone was noninferior to initial CT plus additional MRI with regard to clinical outcomes at discharge and at 1 year.

**Meaning:**

Results of this study suggest that further research is needed to determine which patients hospitalized with acute ischemic stroke benefit from MRI in addition to initial CT.

## Teaser

This cohort study of patients hospitalized with acute ischemic stroke assesses whether a diagnostic imaging strategy of initial computed tomography (CT) alone was noninferior to initial CT with additional magnetic resonance imaging (MRI) with regard to clinical outcomes at discharge and at 1 year.

## Introduction

Unnecessary medical imaging is a major cause of preventable waste in the US health care system.^1,2,3^ Use of computed tomography (CT) and magnetic resonance imaging (MRI) has increased rapidly since 2000 and is greater than in other high-income countries.^2,3^ Most evaluations of medical imaging report diagnostic accuracy or resultant changes in management rather than associations with patient outcomes, with recent publications noting a need for more studies targeted at clinical end points.^4,5,6^ Use of MRI to evaluate patients with acute ischemic stroke (AIS) is a case in point. Between 1999 and 2008, use of MRI increased from 28% to 66% of patients. More than 90% received MRI in addition to CT.^7^ Few data are available about the association between additional MRI and clinical outcomes after stroke to assess this increase in use.^4,8,9^

There are possible reasons why MRI may have added value to initial CT for patients with AIS. The MRI-derived information about stroke subtype, timing, or location may lead to better selection of treatments.^4^ Clear demonstration of infarction has been proposed as a means to enhance patient education and improve adherence to prescribed prevention regimens.^10^

In patients hospitalized with AIS, we combined propensity-score matching with data from an electronic medical record system in a retrospective observational cohort study to assess whether a diagnostic imaging strategy of initial CT alone was noninferior to initial CT with additional MRI with regard to clinical outcomes at discharge and at 1 year.

## Methods

### Study Design

This cohort study used retrospective review of the electronic medical records of patients admitted to the University of North Carolina Hospitals Comprehensive Stroke Center between January 2015 and December 2017. This period was chosen to coincide with variations in espoused attitudes among the attending neurologists with regard to ordering MRI. We did not perform a sample size calculation because we assumed that our sample was restricted to the years 2015 to 2017, during which frequent MRI was espoused by some. We therefore considered this as our time window to propensity score match patients. On review of additional data, we determined that this assumption was incorrect. We reviewed MRI orders to identify the supervising physician and MRI reports to determine the specified indication for the study. This study was approved by the University of North Carolina at Chapel Hill Institutional Review Board, which waived the requirement for informed patient consent due to minimal risk. This report follows the Strengthening the Reporting of Observational Studies in Epidemiology (STROBE) reporting guideline for cohort studies.

Data on the initial hospitalization were obtained from electronic medical records searched from May 2020 through December 2021. For the propensity score–matched groups, outcomes at 1 year after discharge were determined from search of electronic medical records and online obituary postings through January 2022. These follow-up searches were performed by 2 investigators who were not told whether the patients had received MRI or not. However, in the course of the medical record review, they may have seen this information.

Admission diagnosis was made by the neurology resident on call who may have been in the second, third, or fourth year of postgraduate training. Our residency training program does not require discussion with an attending physician at the time of admission but does require second-year residents to discuss with a more senior resident. After admission, patients may remain in the emergency department in the care of an emergency physician while awaiting a bed. Patients who are admitted to the neuroscience intensive care unit received care from nurse practitioners under supervision of neurocritical care physicians. During hospitalization, all patients received care from an attending neurologist who was certified in vascular neurology by the American Board of Psychiatry and Neurology or who had completed a subspecialty residency in vascular neurology accredited by the Accreditation Council for Graduate Medical Education and subsequently was certified in vascular neurology by the American Board of Psychiatry and Neurology. Residents may order MRIs on their own without prior attending consultation.

### Patients

Patients with a discharge diagnosis of AIS were identified from our local Get With The Guidelines stroke database. Inclusion criteria for this study were at least 18 years of age, admission diagnosis of AIS based on neurological evaluation, and initial CT (and before MRI, if performed). Exclusion criteria were MRI performed before admission diagnosis, unclear time of last known normal, unknown modified Rankin Scale score (mRS) at baseline or discharge, unknown National Institutes of Health Stroke Scale (NIHSS) score at admission, or incident AIS occurring in hospital.

### Outcomes

Death or dependence at discharge was defined by mRS scores of 3 to 6. Scores for the mRS range from 0 (no symptoms) to 6 (death). An mRS of 1 indicates ability to carry out all usual duties and activities despite symptoms. An mRS of 2 indicates inability to carry out all normal activities but ability to look after one’s own affairs without assistance. An mRS of 3 indicates needing some help but able to walk unassisted. An mRS of 4 indicates inability to attend to own bodily needs without assistance and unable to walk unassisted. An mRS of 5 indicates requirement for constant nursing care and attention, and being bedridden and incontinent.^11^ Clinical status at 1 year was determined as the composite end point of stroke or death occurring within 12 months after hospital discharge from the index stroke event. Death during hospitalization was also recorded for comparison with a previous study.^9^

### Statistical Analysis

Propensity scores were estimated using a logistic regression model based on the use of MRI as the dependent variable, and the 26 baseline and intervention characteristics listed in Table 1 as covariates. These covariates were selected with reference to published literature.^12,13,14,15,16,17,18,19,20,21^ Propensity-score matching of the 123 patients with additional MRI to 123 patients who did not have additional MRI was conducted by the optimal full-matching approach without replacement using Optmatch.^22^ Optimal full matching is a method of propensity-score matching that does not rely on identification of closely matched pairs. The method is based on grouping individuals into subsets that contain at least 1 treated patient and at least 1 control patient. Treated individuals may be matched with many controls if they have similar propensity scores. Optimal full matching has the advantage of keeping all treated patients in the final analysis cohort, avoiding exclusion of some treated cases because there is no closely matched control pair. The validity of the propensity score–matching model is determined by whether it produces 2 cohorts that are well-matched for the variables.^23^

**Table 1.** Patient Characteristics of All Included Patients

Variable	No. (%)		95% CI^a^	*P* value	SMD
	Patients who underwent MRI (n = 123)	Patients who did not undergo MRI (n = 385)			
Age, median (IQR), y	68.4 (58-78.8)	69.4 (59.9-81)	−4.9 to 1.2	.24	0.142
Sex					
Male	65 (52.8)	220 (57.1)	−14.4 to 5.8	.40	0.086
Female	58 (47.2)	165 (42.9)	−5.8 to 14.4		
LKN to admission, median (IQR), h	7 (3-13)	4.1 (2.5-6.3)	1.3 to 3.4	<.001	0.280
Admission NIHSS, median (IQR)	5 (3-10.5)	8 (4-17)	3 to 1	<.001	0.362
Baseline mRS, median (IQR)	0 (0-1)	0 (0-1)	0 to 0	.60	0.037
Admission serum glucose level, median (IQR), mg/dL	118 (98-148)	118 (100-152)	−7 to 6	.66	0.058
Hypertension	92 (74.8)	299 (77.7)	−11.6 to 5.9	.51	0.067
Hyperlipidemia	63 (51.2)	195 (50.6)	−9.6 to 10.7	.91	0.011
Diabetes	39 (31.7)	119 (30.9)	−8.6 to 10.2	.87	0.017
Coronary disease	17 (13.8)	92 (23.9)	−17.5 to −2.6	.02	0.259
Previous myocardial infarction	7 (5.7)	32 (8.3)	−7.6 to 2.3	.34	0.103
Atrial fibrillation	19 (15.4)	110 (28.6)	−20.9 to −5.3	.004	0.320
Heart failure	3 (2.4)	50 (13)	−14.9 to −6.2	.001	0.403
Peripheral artery disease	11 (8.9)	33 (8.6)	−5.4 to 6.1	.90	0.013
Chronic kidney disease	12 (9.0)	29 (7.5)	−3.6 to 8.1	.43	0.079
Obstructive sleep apnea	6 (4.9)	25 (6.5)	−6.1 to 2.9	.52	0.070
Previous stroke	35 (28.5)	91 (23.6)	−4.2 to 13.9	.28	0.110
Previous TIA	6 (4.9)	27 (7)	−6.7 to 2.4	.40	0.090
BMI, median (IQR)	28.4 (24.4-32.6)	27.7 (23.7-32.1)	−0.5 to 2	.23	0.168
Malignant neoplasm	20 (16.3)	52 (13.5)	−4.6 to 10.1	.47	0.077
Current smoking	28 (22.8)	89 (23.1)	−8.9 to 8.2	.94	0.008
Statin pretreatment	54 (43.9)	199 (43.1)	−9.3 to 10.9	.88	0.016
CCI, median (IQR)	4 (2-5)	4 (2-6)	−1 to 0	.37	0.115
CT with acute ischemic changes	24 (19.5)	120 (31.2)	−20.1 to −3.3	.01	0.270
Intravenous thrombolysis	35 (28.5)	229 (59)	−39.9 to −21.1	<.001	0.645
Endovascular treatment	8 (6.5)	89 (23.1)	−22.7 to −10.6	<.001	0.480

Abbreviations: BMI, body mass index (calculated as weight in kilograms divided by height in meters squared); CCI, Charlson Comorbidity Index; CT, computed tomography; LKN, last known normal; MRI, magnetic resonance imaging; mRS, modified Rankin Scale score; NIHSS, NIH Stroke Scale; SMD, standardized mean difference; TIA, transient ischemic attack.

SI conversion factor: To convert serum glucose to millimoles per liter, multiply by 0.0555.

^a^ The 95% CI for the difference between medians or percentages subtracting patients who did not undergo MRI from patients who underwent MRI.

Paired comparisons of covariates before matching and after matching were performed with the 2-proportion *z* test for categorical variables, and either Wilcoxon rank sum test/Mann-Whitney test or *t* test for quantitative variables. Shapiro-Wilk testing was used to assess for normality assumption violation. Standardized mean difference (SMD) was estimated to assess covariate balance before matching and after matching. An SMD less than 0.100 for any given covariate indicated satisfactory balance between groups. The *P* values are reported without correction for increased type I error due to multiple analyses and should be interpreted with this limitation.

Noninferiority margins for the 2 clinical outcomes were based on the design characteristics of previous randomized clinical trials of stroke treatment, which were identified before the results of this study were known. Two noninferiority trials of mechanical thrombectomy with or without alteplase that used the percentage of patients with an mRS score of 0 to 2 at 3 months as the primary outcome measure designated noninferiority margins of 5% or 10%.^24,25^ We used the mean value of −7.5% for our noninferiority margin for the percentage of patients discharged with an mRS score of 3 to 6 because the 2 ranges are complementary. Two previous randomized trials of long-term secondary stroke prevention that used stroke or death rates as the primary outcome designated end point reductions of 25% or 30% as the minimum important clinical benefit in their power and sample size calculations.^26,27^ We used the mean value of a 27.5% corresponding to a relative risk of 0.725 to set our noninferiority margin. These 2 studies provided data on total events per unit time, not time-to-event data. Thus, we computed relative risks (not hazard ratios) for our noninferiority analysis. To declare noninferiority, we required a 1-sided significance level of .025 as demonstrated by the lower bound of the 95% CIs excluding the noninferiority margins.

A 2-sided χ^2^ test was performed to compare the outcome of hospital mortality. This *P* value is reported without correction for increased type I error due to multiple analyses and should be interpreted with this limitation. Patients lost to follow-up after discharge were not included in the 1-year analysis. All statistical analyses were performed using R, version 4.1.2 (R Foundation for Statistical Computing). The statistical analysis was performed in January 2022.

## Results

### Study Cohort

Among the final cohort of 246 propensity score–matched participants, the median age was 68 years (IQR, 58-79 years) and 131 (53%) were men. We initially identified 508 patients who met eligibility criteria (Figure). There were 123 patients who had additional MRI and 385 patients who had CT alone. Characteristics of these patients are shown in Table 1. Thirteen of 26 covariates were unbalanced between groups by an SMD greater than or equal to 0.100. CT findings of acute ischemia were noted in the neuroradiologist report in 19.5% of those with additional MRI and in 31.2% of those with initial CT only. Propensity-score matching paired all 123 patients with additional MRI to 123 patients with initial CT alone (Figure). An SMD greater than or equal to 0.100 remained only for history of coronary artery disease (SMD = 0.111) and chronic kidney disease (SMD = 0.119) (Table 2) (eFigure 1 in the Supplement).

**Figure.**
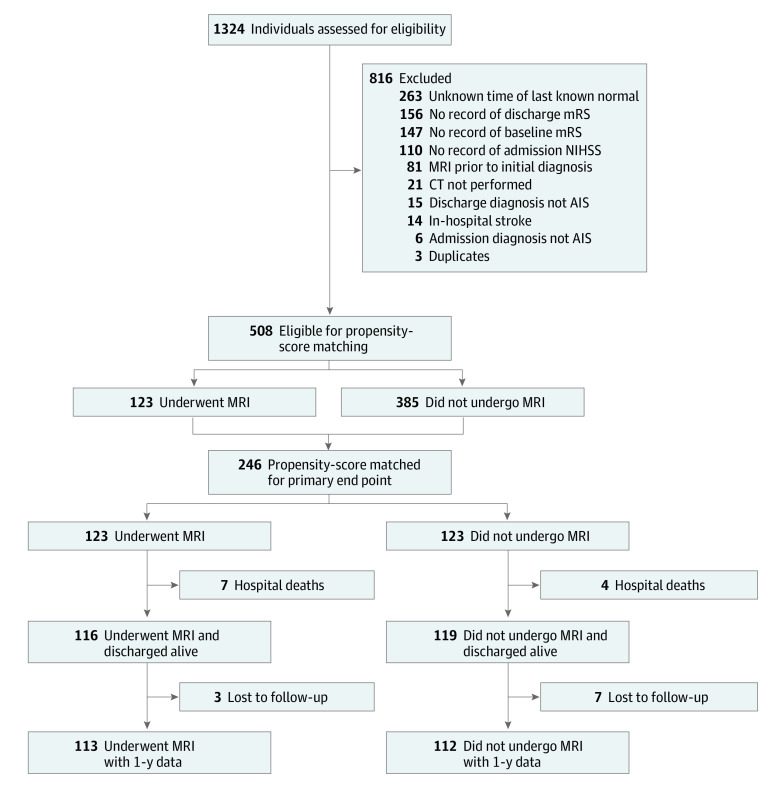
Participant Flow Diagram AIS indicates acute ischemic stroke; CT, computed tomography; MRI, magnetic resonance imaging; mRS, modified Rankin Scale score; NIHSS; National Institutes of Health Stroke Scale.

**Table 2.** Patient Characteristics of the Propensity Score–Matched Cohorts

Variable	No. (%)		95% CI^a^	*P* value	SMD
	Patients who underwent MRI (n = 123)	Patients who did not undergo MRI (n = 123)			
Age, median (IQR), y	68.4 (58-78.8)	68 (58.2-79.1)	−3.7 to 3.5	.99	0.034
Sex					
Male	65 (52.8)	66 (53.7)	−13.3 to 11.7	.90	0.016
Female	58 (47.2)	57 (46.3)	−11.7 to 13.3		
LKN to admission, hours, median (IQR)	7 (3.0-13)	4.9 (2.9-10.4)	−0.2 to 2.4	.12	0.005
Admission NIHSS, median (IQR)	5 (3.0-10.5)	5 (3-11)	−1 to 1	.88	0.024
Baseline mRS, median (IQR)	0 (0-1)	0 (0-1)	−0 to 0	.36	0.045
Admission serum glucose level, median (IQR), mg/dL	118 (98-148)	113 (101-143)	−6 to 9	.64	0.025
Hypertension	92 (74.8)	96 (78)	−13.9 to 7.3	.55	0.076
Hyperlipidemia	63 (51.2)	64 (52)	−13.3 to 11.7	.90	0.016
Diabetes	39 (31.7)	34 (27.6)	−7.3 to 15.5	.49	0.089
Coronary disease	17 (13.8)	22 (17.9)	−13.2 to 5.0	.38	0.111
Previous myocardial infarction	7 (5.7)	7 (5.7)	−5.8 to 5.8	>.99	0.000
Atrial fibrillation	19 (15.4)	17 (13.8)	−7.2 to 10.5	.72	0.046
Heart failure	3 (2.4)	3 (2.4)	−3.9 to 3.9	>.99	0.000
Peripheral artery disease	11 (8.9)	11 (8.9)	−7.1 to 7.1	>.99	0.000
Chronic kidney disease	12 (9.0)	8 (6.5)	−3.6 to 10.1	.35	0.119
Obstructive sleep apnea	6 (4.9)	4 (3.3)	−3.3 to 6,6	.52	0.082
Previous stroke	35 (28.5)	31 (25.2)	−7.8 to 14.3	.57	0.073
Previous TIA	6 (4.9)	6 (4.9)	−5.4 to 5.4	>.99	0.000
BMI, median (IQR)	28.4 (24.4-32.6)	27.9 (24.1-33.3)	−1.4 to 1.9	.73	0.058
Malignant neoplasm	20 (16.3)	22 (17.9)	−11.0 to 7.8	.74	0.043
Current smoking	28 (22.8)	28 (22.8)	−10.5 to 10.5	>.99	0.000
Statin pretreatment	54 (43.9)	53 (43.1)	−11.6 to 13.2	.90	0.016
CCI, median (IQR)	4 (2-5)	3 (2-5)	−1 to 1	.83	0.023
CT with acute ischemic changes	24 (19.5)	29 (23.6)	−14.3 to 6.2	.44	0.099
Intravenous thrombolysis	35 (28.5)	40 (32.5)	−15.6 to 7.4	.49	0.088
Endovascular treatment	8 (6.5)	8 (6.5)	−6.1 to 6.1	>.99	0.000

Abbreviations: BMI, body mass index (calculated as weight in kilograms divided by height in meters squared); CCI, Charlson Comorbidity Index; CT, computed tomography; LKN, last known normal; MRI; magnetic resonance imaging; mRS, modified Rankin Scale score; NIHSS, NIH Stroke Scale; SMD, standardized mean difference; TIA, transient ischemic attack.

SI conversion factor: To convert serum glucose to millimoles per liter, multiply by 0.0555.

^a^ The 95% CI for the difference between medians or percentages subtracting patients who did not undergo MRI from patients who underwent MRI.

### Magnetic Resonance Imaging

Of the 123 MRIs, 52 (42.3%) were ordered under the supervision of attending vascular neurologists, 41 (33.3%) under supervision of attending emergency physicians, and 30 (24.4%) by nurse practitioners or neurocritical care attending physicians while patients were in the neuroscience intensive care unit. Orders for MRI were evenly distributed across 6 attending vascular neurologists when adjusted for time on service. This distribution may reflect residents ordering MRIs on their own without prior attending consultation. The MRIs ordered by emergency physicians were performed after the neurology resident admission notes were written. Most (26 of 30) MRIs in the neurosciences intensive care unit were ordered during routine care for 24 hours after intravenous thrombolysis.

For 111 of the 123 MRIs, there was no specified indication other than stroke or neurological symptoms. Indications for the remaining 12 MRIs are given in the eTable in the Supplement.

### Outcomes

Death or dependence at discharge occurred more often in patients with additional MRI (59 of 123 [48.0%]) than in those with CT alone (52 of 123 [42.3%]; absolute difference, 5.7%; 95% CI, −6.7% to 18.1%), meeting the −7.50% criterion for noninferiority. Death during hospitalization occurred in 11 patients, 4 of 123 (3.3%) of those who had CT alone and 7 of 123 (5.7%) of those with additional MRI (absolute difference, 2.4%; 95% CI, −2.7% to 7.6%; *P* = .54). Follow-up information to determine stroke or death within 12 months of discharge could be determined for 225 of 235 (96%) patients discharged alive. Stroke or death within 1 year after discharge occurred more often in patients with additional MRI (22 of 113 [19.5%]) than in those with CT alone (14 of 112 [12.5%]; relative risk, 1.14; 95% CI, 0.86-1.50), meeting the 0.725 criterion for noninferiority. The lower bound of the 95% CI for the absolute difference was −2.6% (Table 3). eFigure 2 in Supplement presents Kaplan-Meier time-to-event curves.

**Table 3.** Outcomes in the Propensity Score–Matched Cohorts

Outcome	Group, No./total No. (%)		*P* value	Relative risk (95% CI)	Difference (95% CI)
	Patients who underwent MRI	Patients who did not undergo MRI			
Primary end point					
mRS score 3-6 at discharge	59/123 (48.0)	52/123 (42.3)	.44	1.50 (0.86 to 1.05)	5.7 (−6.7 to 18.1)
Secondary end point					
Stroke or death during 1 y after discharge	22/113 (19.5)	14/112 (12.5)	.23	1.56 (0.84 to 2.89)	6.9 (−2.6 to 16.5)
Death during hospitalization	7/123 (5.7)	4/123 (3.3)	.54	1.75 (0.52 to 5.83)	2.4 (−2.7 to 7.6)

Abbreviations: MRI, magnetic resonance imaging; mRS, modified Rankin Scale.

## Discussion

More than 90% of patients with AIS receive MRI in addition to CT with few data to determine whether there is an associated benefit with patient outcomes.^4,7,8,9^ In this retrospective observational propensity score–matched cohort study of patients hospitalized with AIS, we found that a diagnostic imaging strategy of initial CT alone was noninferior to initial CT with additional MRI with regard to the clinical outcomes of death or dependence at hospital discharge or prevention of stroke or death at 1 year after discharge.

There is no consensus on the methods for selecting noninferiority margins.^28,29^ To avoid bias, we based our selection on data from randomized clinical trials of stroke treatment. Setting a minimally important clinical difference is an important factor in the design of randomized clinical treatment trials. For noninferiority trials, concluding that 2 treatments are not meaningfully different requires demonstration of a low probability that the difference in outcomes was greater than the minimally important clinical difference. Trials to evaluate superiority of one treatment over another are designed to have adequate power to exclude differences equal to or greater than the minimally important clinical difference. We used minimally important clinical differences from the design of randomized clinical treatment trials with the same outcome measures to set our noninferiority margins. Most importantly, these studies were identified before the results of our study were known to us.

Two previous studies have evaluated the association of MRI on outcomes of patient hospitalized with AIS. Hefzy et al^8^ analyzed a prospectively collected cohort of 727 patients with discharge diagnosis of ischemic stroke or transient ischemic attack to determine which baseline clinical variables and diagnostic studies obtained during hospitalization were associated with clinical outcomes at 1 year. None of CT only, CT plus MRI, magnetic resonance angiography alone, echocardiography, or transcranial Doppler was significantly associated with clinical outcomes by multivariate analysis. CT angiography was associated with improvement in outcomes. There were too few patients with MRI alone to analyze. Age and admission NIHSS were factors associated with outcomes. Hefzy et al^8^ concluded that clinical outcomes at 1 year may not be associated with imaging strategies.^8^ In contrast, Lee et al^9^ compared 94 003 patients from the National Inpatient Sample hospitalized with discharge diagnosis of AIS who received MRI during hospitalization to 1 583 768 who did not. Using multivariate analysis to adjust for comorbidities, MRI was associated with lower inpatient mortality (1.67% vs 3.09%; adjusted odds ratio [OR], 0.60; 95% CI, 0.53–0.68; *P* < .001). However, because of the nature of the National Inpatient Sample database, this analysis did not include information on key individual patient baseline variables associated with inpatient mortality including NIHSS score.^16^

Our data are consistent with the conclusions of Hefzy et al^8^ and of Wardlaw et al^30^ who concluded from systematic review, meta-analysis and economic evaluation that the use of MRI in addition to CT was not justified in patients with TIA or minor stroke at a usage frequency of 50%. In contrast with Lee et al,^9^ our data from cohorts matched for baseline variables do not suggest that performance of MRI in addition to CT was associated with improved rates of patient mortality.

### Strengths and Limitations

This study has several strengths. We used clinical data from an electronic medical record system to evaluate routine delivery of medical care. We applied propensity-score matching to adjust for imbalances in baseline characteristics, resulting in 2 well-matched groups. We included all of the patients with additional MRI in our propensity score–matched cohort. We were able to determine follow-up data on 96% of survivors at 1 year after discharge. We report important clinical end points of functional status at hospital discharge and recurrent stroke or death at 12 months.

This study also has limitations. Our data strictly apply to the use of MRI in addition to an initial CT in patients hospitalized with AIS and not to other situations for which MRI may be used, such as choice of initial imaging, transient ischemic attacks, uncertain diagnoses, and in persons awakening with stroke symptoms.^31,32,33,34,35,36^ Data are from a single tertiary referral academic medical center with continuous coverage by in-house neurology residents and subspecialty-trained vascular neurologists. Our findings may not be generalizable to other settings. Only a few patients underwent endovascular treatment, so the results may not be applicable to patients who underwent this treatment. Propensity-score matching may not have completely compensated for relevant baseline differences between the groups if there were important unmeasured confounders. With a retrospective nonrandomized observational cohort study design, this study may be liable to bias. The investigators could not be completely blinded to the performance of MRI during medical record review. Our study does not address whether there is an increase in diagnostic certainty with MRI that affects patients’ sense of well-being.

## Conclusions

In this cohort study, we used propensity-score matching and clinical data from an electronic medical record system to assess outcomes of patients hospitalized with AIS who either did or did not have subsequent MRI in addition to admission CT. Applying noninferiority margins derived from previous randomized clinical trials of ischemic stroke treatments to this cohort, we found that a diagnostic imaging strategy of initial CT alone was noninferior to initial CT with additional MRI with regard to clinical outcomes at discharge and at 1 year. The value of MRI added to CT in patients such as these should not be presumed. Further research is warranted to help determine which patients hospitalized with AIS may benefit from MRI.

## Notes

Supplement. **eFigure 1.** Distribution of Propensity Scores **eTable.** Indications for MRI Other Than Stroke or Neurological Symptoms **eFigure 2.** Kaplan-Meier Curves for the Outcome of Stroke or Death Within 1 Year After Discharge
