# Supplementary material for: Comparison of Outcomes of Ischemic Stroke Initially Imaged With Cranial Computed Tomography Alone vs Computed Tomography Plus Magnetic Resonance Imaging
Source: JAMA Netw Open. 2022 Jul 21;5(7):e2219416. doi: 10.1001/jamanetworkopen.2022.19416 (PMC9305377; doi:10.1001/jamanetworkopen.2022.19416)
Supplement: Supplement. — eFigure 1. Distribution of Propensity Scores eTable. Indications for MRI Other Than Stroke or Neurological Symptoms eFigure 2. Kaplan-Meier Curves for the Outcome of Stroke or Death Within 1 Year After Discharge [file jamanetwopen-e2219416-s001.pdf]

Supplemental Online Content

Cabral Frade H, Wilson SE, Beckwith A, Powers WJ. Comparison of outcomes of ischemic stroke initially imaged with cranial computed tomography alone vs computed tomography plus magnetic resonance imaging. *JAMA Netw Open.* 2022;5(7):e2219416. doi:10.1001/jamanetworkopen.2022.19416

eFigure 1. Distribution of Propensity Scores

eTable. Indications for MRI Other Than Stroke or Neurological Symptoms

eFigure 2. Kaplan-Meier Curves for the Outcome of Stroke or Death Within 1 Year After Discharge

This supplemental material has been provided by the authors to give readers additional information about their work.

© 2022 Cabral Frade H et al. *JAMA Network Open*.

eFigure 1
Distribution of propensity scores
Treated Units are participants with MRI. Control Units are participant without MRI.

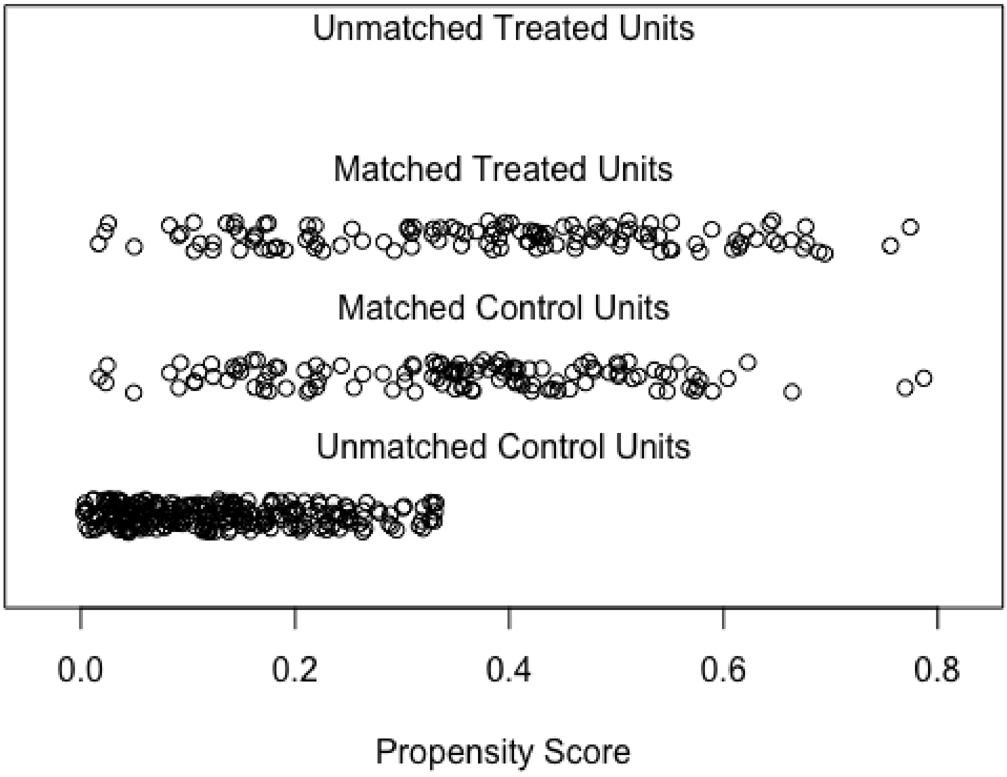

© 2022 Cabral Frade H et al. *JAMA Network Open*.

eTable
Indications for MRI other than stroke or neurological symptoms

68 year old M. Left sided weakness and slurred speech. Evaluate for stroke in patient with pituitary mass.
80 Year Old (F): SEIZURES/ CONVULSIONS.
58 year-old M with a history of stroke and cerebellar hemorrhage. Patient presents with sudden onset headache.
82 Year Old (F): Stroke versus mass on head CT
58 years old Female with stroke-- reported history of "MS like syndrome ", with stroke symptoms beginning 8/11/2017, transferred for further workup and treatment.
63 years old Male with stroke v PRES-- previous history of old right MCA infarction, currently with clinical concern for new infarction
66 years old Female with stroke in setting of possible TTP--
75 years old Female with CVA, query possible mets as well--
85 Year Old (M) with stroke and intracranial hemorrhage
56 Year Old (M): Evaluate for stroke, seizure, and brain metastases. History of stage IV colon cancer.
81 year-old M with history of left parietal lobe infarct and corpus callosum hemorrhage.
49 years old Male with stroke-- seizure activity, recent onset left-sided weakness

© 2022 Cabral Frade H et al. *JAMA Network Open*.

eFigure 2
Kaplan-Meier curves for the outcome of stroke or death within 1 year after discharge

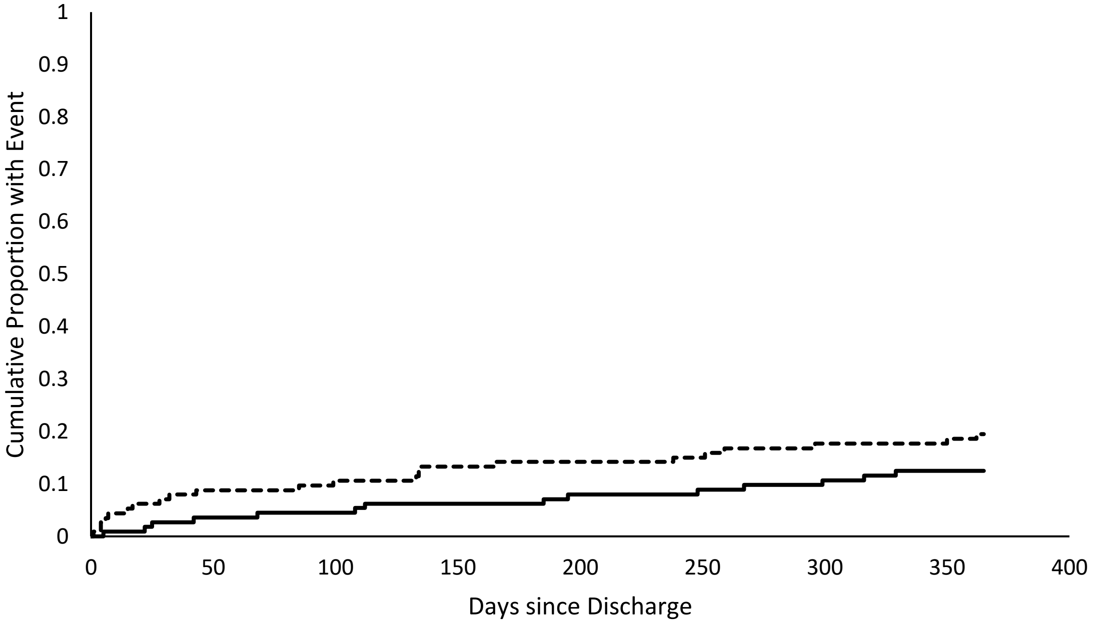

Dashed line - Participants with MRI. Solid line – participants without MRI.

© 2022 Cabral Frade H et al. *JAMA Network Open*.
